# LEARNING LIFE COURSE CONCEPTS THROUGH FILM

**DOI:** 10.1093/geroni/igz038.3068

**Published:** 2019-11-08

**Authors:** Lisa C Hall

**Affiliations:** Missouri State University, Springfield, Missouri, United States

## Abstract

This paper explains how students learned foundational concepts through viewing a commercial film. The students (mostly social, behavioral, and biomedical sciences majors) were enrolled in a survey course for the gerontology major in a Program of Merit-certified Bachelor of Science program. Elements of the life course (transitions, counter-transitions, trajectory, age norms and timetables, sequence, duration, and social clock), and types of aging (biological, psychological, social, chronological, functional and subjective) are evident in the 2008 fictional film, The Curious Case of Benjamin Button. An adaptation of F. Scott Fitzgerald’s short story by the same name, the film chronicles a man’s life who was born physiologically old and dies as an infant with dementia. A multi-phased, active learning approach was used in which students (1) read about the abstract concepts from a textbook, (2) viewed the film while simultaneously identifying and applying concepts through notetaking, reflective writing prompts, and class discussion, (3) defined concepts and critically analyzed the contents in a written assessment, and (4) recalled definitions of concepts, eight weeks later, in a multiple choice final exam. Over three-quarters of students scored 80% or higher on the written assessment. Students under the active learning approach demonstrated higher recall on the final exam compared to students who were under the previous approach of standard lecture. Though these findings indicate that active learning through film is effective, they were realized retrospectively while attempting to improve a course’s learning outcomes. A planned, cross-sectional study could better account for variability and yield more conclusive results.

